# The Seamless Transfer-of-Care Protocol: a randomized controlled trial assessing the efficacy of an electronic transfer-of-care communication tool

**DOI:** 10.1186/1472-6963-12-414

**Published:** 2012-11-21

**Authors:** Barbara M Okoniewska, Maria J Santana, Jayna Holroyd-Leduc, Ward Flemons, Maeve O’Beirne, Deborah White, Fiona Clement, Alan Forster, William A Ghali

**Affiliations:** 1 Department of Community Health Sciences, W21C Research and Innovation Centre, Institute of Public Health, University of Calgary, 3280 Hospital Drive NW, Calgary, AB, Canada; 2 Department of Community Health Sciences, Faculty of Medicine, University of Calgary, 3280 Hospital Drive NW, Calgary, AB, Canada; 3 Department of Medicine, Faculty of Medicine, University of Calgary, 3280 Hospital Drive NW, Calgary, AB, Canada; 4 Department of Medicine, Faculty of Medicine, University of Calgary, 3280 Hospital Drive NW, Calgary, AB, Canada; 5 Department of Family Medicine, Faculty of Medicine, University of Calgary, 3280 Hospital Drive NW, Calgary, AB, Canada; 6 Faculty of Nursing, University of Calgary, 3280 Hospital Drive NW, Calgary, AB, Canada; 7 Department of Medicine, University of Ottawa, Ottawa, Canada

**Keywords:** Medical informatics, Care transitions, Electronic health records, Randomized controlled trials, Hospital discharge

## Abstract

**Background:**

The transition between acute care and community care represents a vulnerable period in health care delivery. The vulnerability of this period has been attributed to changes to patients’ medication regimens during hospitalization, failure to reconcile discrepancies between admission and discharge and the burdening of patients/families to take over care responsibilities at discharge and to relay important information to the primary care physician. Electronic communication platforms can provide an immediate link between acute care and community care physicians (and other community providers), designed to ensure consistent information transfer. This study examines whether a transfer-of-care (TOC) communication tool is efficacious and cost-effective for reducing hospital readmission, adverse events and adverse drug events as well as reducing death.

**Methods:**

A randomized controlled trial conducted on the Medical Teaching Unit of a Canadian tertiary care centre will evaluate the efficacy and cost-effectiveness of a TOC communication tool. Medical in-patients admitted to the unit will be considered for this study. Data will be collected upon admission, and a total of 1400 patients will be randomized. The control group’s acute care stay will be summarized using a traditional dictated summary, while the intervention group will have a summary generated using the TOC communication tool. The primary outcome will be a composite, at 3 months, of death or readmission to any Alberta acute-care hospital. Secondary outcomes will be the occurrence of post-discharge adverse events and adverse drug events at 1 month post discharge. Patients with adverse outcomes will have their cases reviewed by two Royal College certified internists or College-certified family physicians, blinded to patients’ group assignments, to determine the type, severity, preventability and ameliorability of all detected adverse outcomes. An accompanying economic evaluation will assess the cost per life saved, cost per readmission avoided and cost per QALY gained with the TOC communication tool compared to traditional dictation summaries.

**Discussion:**

This paper outlines the study protocol for a randomized controlled trial evaluating an electronic transfer-of-care communication tool, with sufficient statistical power to assess the impact of the tool on the significant outcomes of post-discharge death or readmission. The study findings will inform health systems around the world on the potential benefits of such tools, and the value for money associated with their widespread implementation.

**Trial registration:**

ClinicalTrials.gov NCT01402609.

## Background

The transition between acute care and community care represents one of the most vulnerable periods in health care delivery, particularly as the complexity of inpatient populations increases. The vulnerability of this period has been attributed to three main factors. First, changes to patients’ medication regimens during hospitalization are numerous, yet failure to reconcile discrepancies between admission and discharge is frequent [1]. Second, the patient/family is required to take over care responsibilities at discharge and must often personally relay important information to the primary care physician [1]. This can be particularly challenging if the TOC information is poorly communicated, presented too rapidly, if instructions are verbal only, or if the patient struggles with health literacy [1-3]. Finally, crucial information is often not transferred between acute care physicians and community physicians [1].

Information about the hospitalization (such as medication changes, patient diagnoses, interventions, diagnostic findings, and necessary follow-up) is commonly transferred to the community care physician through a summary of the patient’s acute care stay that is faxed or mailed. Deficits with respect to timeliness and/or complete failure to transmit are widespread [1,4]. At the first post-discharge appointment, this summary is unavailable to the community care physicians up to 75% of the time [1,5-7]. This negatively impacts the continuity of care provided to many patients [1,7]. When summaries are received, inconsistent content and inaccuracies are common [1,4]. Acute care physicians, whether medical or surgical, often neglect to include diagnostic findings, treatment/hospital course, discharge medications, pending tests results, and whether the patient and family received counseling [7].

Computer-enabled TOC communications have potential to avert such problems. These communication tools, operating on electronic health record or web-based platforms can provide an immediate link between acute care and primary care physicians, and interfaces can be designed to ensure consistent information transfer. In addition, physicians in both settings have expressed preference for electronic discharge documents over hand written/dictated summaries with respect to clarity, comprehensiveness, and positive impacts on continuity of care [8-11].

Kripalani and collegues [4] published a systematic review examining the prevalence of discharge communication deficits and looked broadly at all types of interventions that target those deficits. Very few of the interventions reviewed by Kripilani et al. involved significant contributions from electronic medical record data to construct the discharge summary, and none used the internet to transmit information. However, the publications years considered spanned 1977 to 2005.

In recent years, original studies [9-20] have emerged assessing the efficacy of computer enabled TOC communication compared to traditional summaries. In the context of a rapidly growing literature on electronic medical records and telehealth interventions, our team systematically reviewed the literature and identified 12 controlled studies assessing the efficacy of computer-enabled TOC communication tools [14]. The findings in the literature globally indicate that compared to traditional TOC summaries, computer-enabled TOC communications of various types appear promising, particularly with respect to improving timeliness of discharge summary delivery, and satisfaction among physicians and patients/families. There are also some positive impacts on overall patient management and continuity of care. However, only four of these studies reported the effects of such tools on the notable clinical endpoints of hospital readmission, post-discharge mortality, and adverse events [14], and in doing so, none had sufficient statistical power to assess these endpoints. As a result, only one study [15] in our review demonstrated a reduction in readmission to hospital within 12 months due to the implementation of a web-based TOC communication platform. Our published systematic review identified a need for a well-designed, sufficiently powered study evaluating relevant clinical outcomes associated with an electronically based TOC tool. Given the growing demand and monetary investments associated with these tools, such a study would have implications on a global scale.

The Ward of the 21^st^ century (W21C) initiative at the University of Calgary, in Alberta, Canada is an interdisciplinary research and innovation program focused on health system safety and quality (see http://www.w21c.org). The W21C team, working collaboratively with Alberta Health Services, the provincial health authority, undertook iterative consultation with multiple clinical stakeholders as well as patients and family, and developed an electronic communication TOC tool. The tool was built off of the already functioning electronic platform called Sunrise Clinical Manager (SCM) that physicians use to manage inpatient orders and to access patient medical records, diagnostic imaging and laboratory results. An initial pilot test of the tool involving 100 actual patient transfers of care summaries from an adult internal medicine ward to the community was performed on the Medical Teaching Unit (MTU) affiliated with the W21C. This pilot study involved detailed assessments of usability issues, provider and patient satisfaction, completeness and timeliness of the discharge summaries. The information generated from this pilot led to the refinement of the tool to the extent that it satisfied the needs of acute care physicians, primary care physicians, and patients. The refined tool will be made available for use by physicians and medical students on the MTU and used for this study.

On this backdrop of iterative tool development and pilot work, a protocol for a randomized controlled trial to more definitely test the efficacy of the TOC communication tool was developed. With funding from the Canadian Institutes of Health Research, the RCT will assess the efficacy of the TOC communication tool for reducing the endpoint of death or hospital readmission at 3 months. Secondary objectives are: i) to assess the impact of the tool on the occurrence of post-discharge adverse events and adverse drug events; ii) to evaluate the cost-effectiveness of the tool.

## Methods

### Setting

The study will be conducted on the Medical Teaching Unit (MTU) of a Canadian tertiary Medical Center. The MTU is a general internal medicine unit that provides care to adults who have non-surgical medical problems that necessitate hospitalization. The typical patient on the team has multi-system disease and/or complex symptom presentations that require detailed diagnostic evaluation and/or inpatient therapies. Patients typically have multiple comorbid illnesses and complicated medication profiles that require careful scrutiny and review at time of discharge. Pilot work on the MTU revealed that approximately one in four patients discharged from the unit were readmitted within a 3 month period (i.e., 51 out of 219 cases reviewed in our pilot work).

The MTU consists of three physician teams working in parallel on multiple hospital wards throughout the hospital. The three teams have indistinguishable clinical expertise and consist of similarly-trained attending staff physicians and rotating medical residents and students. They care for similar patient populations and work in the same environment, with assistance from the shared nurse discharge coordinator. The teams are already undertaking a diligent process of pre-acute care discharge preparation (i.e., prior to implementation of the TOC communication tool) in which attempts are made to prepare communication materials for community providers. This is therefore a highly appropriate setting for the study of a TOC tool, because the patient population is at high risk for readmission, and one for whom enhanced clinical communication produced by the TOC communication tool can be evaluated on a backdrop of already diligent discharge preparation practices.

### Participants

All patients admitted to the MTU will be considered for this study. Patients will be excluded if the patient and/or family member declines consent; is under 18 years of age; cannot provide contact information; and/or family member lacks English proficiency and the team cannot communicate with them; has a research burden (enrolled in 2 other studies); is admitted under or has their acute care transferred to a clinical service other than the MTU; is not an Alberta resident; was previously enrolled in the study; is being discharged to hospice care; is transferred to another Hospital (“Rapid Transport”); is incoherent; or dies in hospital.

Written information about the study will be provided to patients before obtaining informed consent. The study has been approved by the Conjoint Health Research Ethics Board at the University of Calgary (# 23469) and registered at ClinicalTrials.gov (NCT01402609).

### Study design

**Figure 1:**
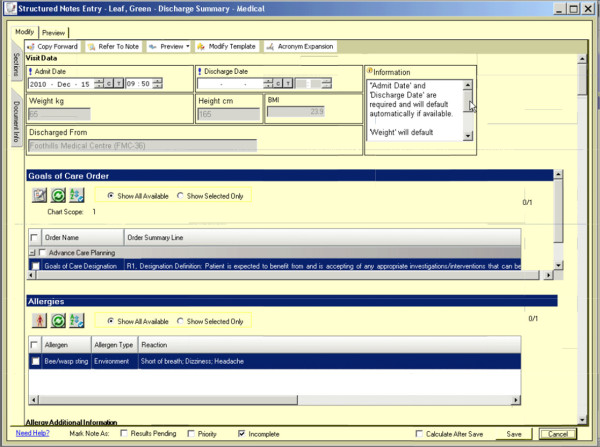
The introduction page for a test patient from the seamless transfer-of-care tool.

**Figure 2:**
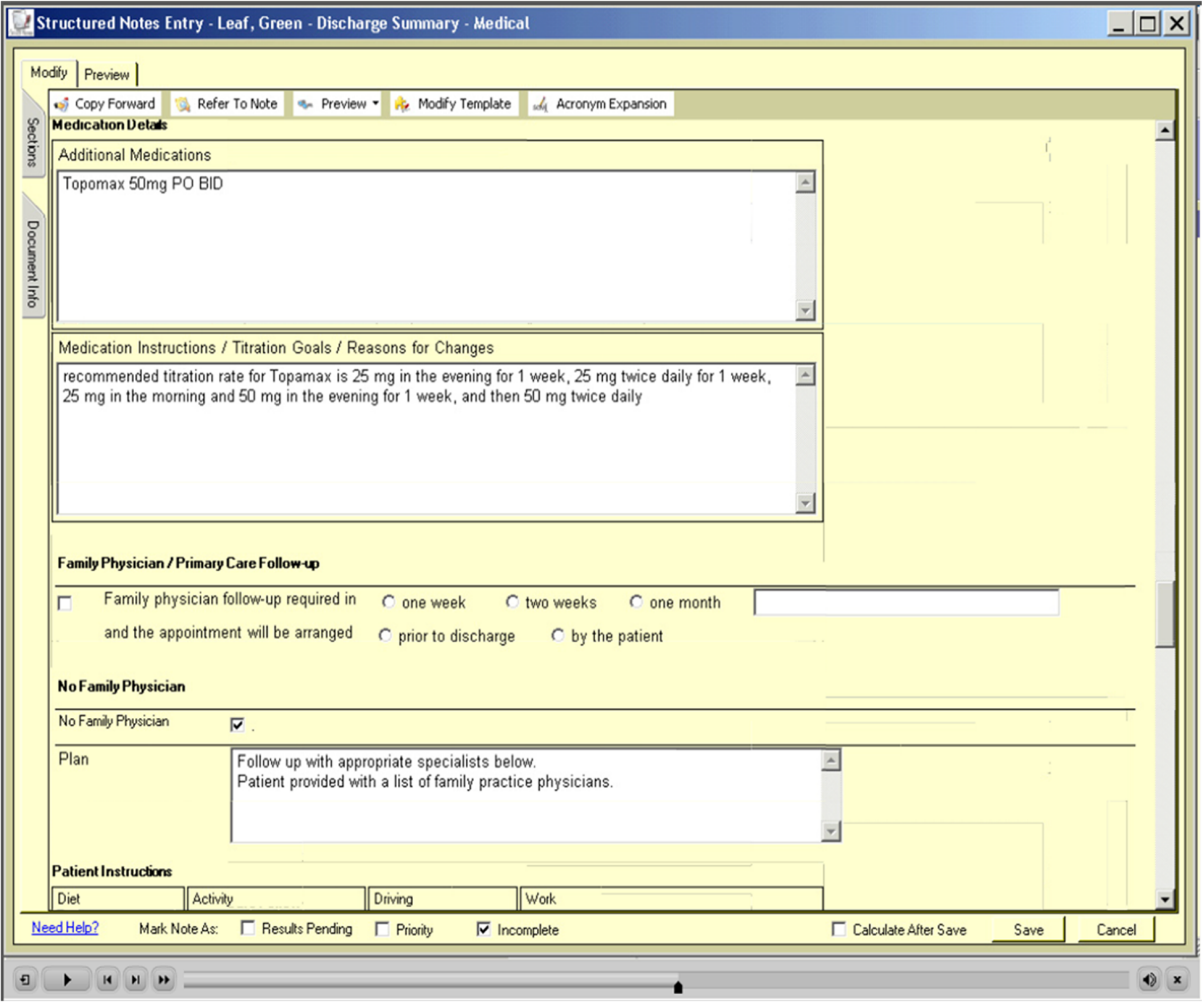
The medication details off of a test patient from the seamless transfer-of-care tool.

**Figure 3:**
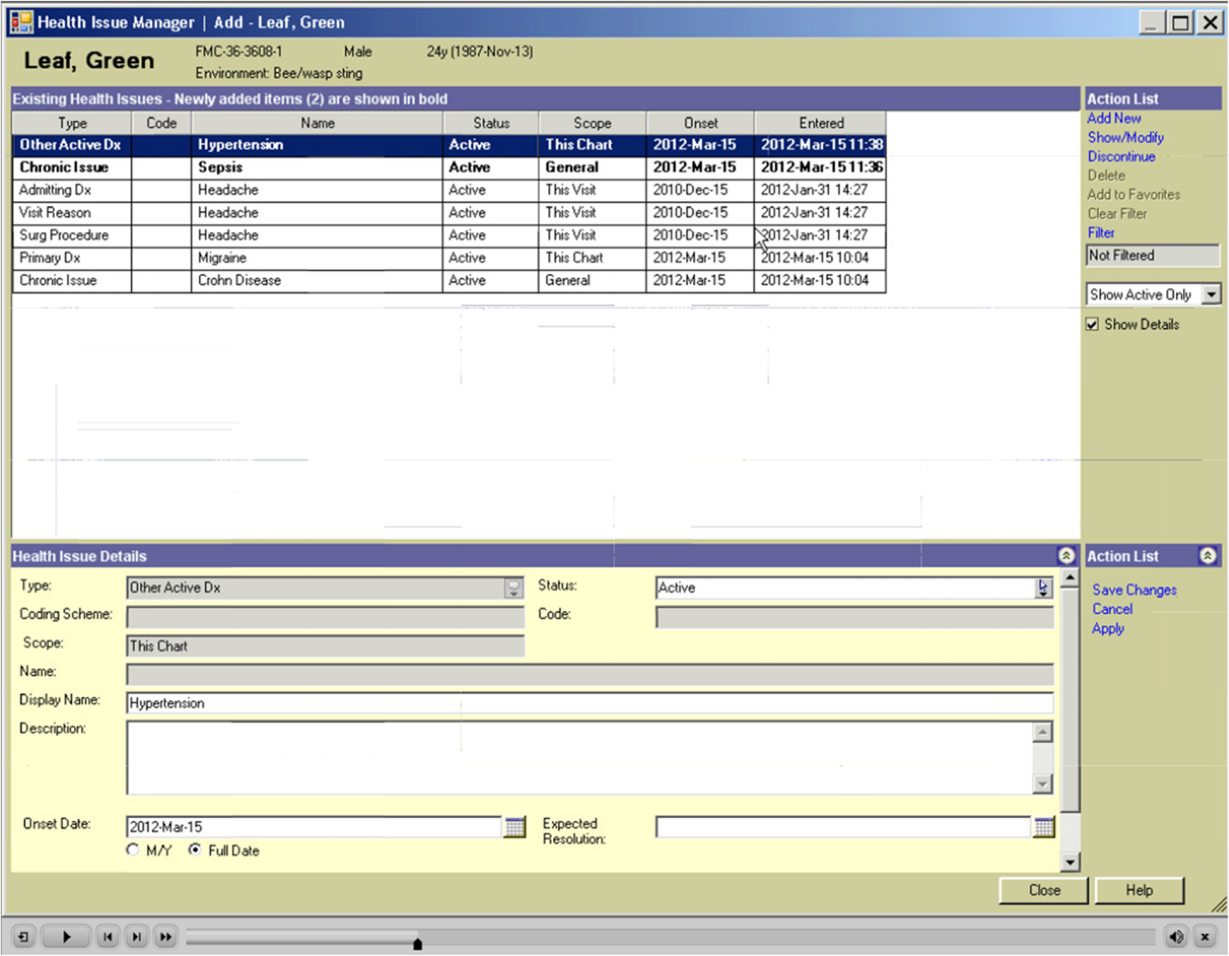
Existing health issues off of a test patient from the seamless transfer-of-care tool.

Patients will be randomly assigned to discharge from acute care with the TOC tool (intervention group) *versus* usual care (control group). The intervention will consist of patient’s acute-care stay summaries being generated with the use of the computer-enabled communication tool. The tool provides a standardized template for communicating all relevant clinical information on patients who are leaving hospital, is immediately available at time of leaving and is accessible through the web by community-based providers (family physicians, specialists, home care nurses, and community pharmacists). Figure 1, Figure 2, Figure 3 show screen shots taken from various points in the TOC tool. This is in contrast to current summaries that the control group will receive under usual care (control group). Usual care involves using the traditional model of TOC communication with dictated/transcribed summaries. This usual care typically involves paper-based handwritten summaries with subsequent provision of a dictated summary produced some time after the patient leaves hospital, with unpredictable success and timing of delivery and with unstructured and sometimes haphazard content.

**Figure 4:**
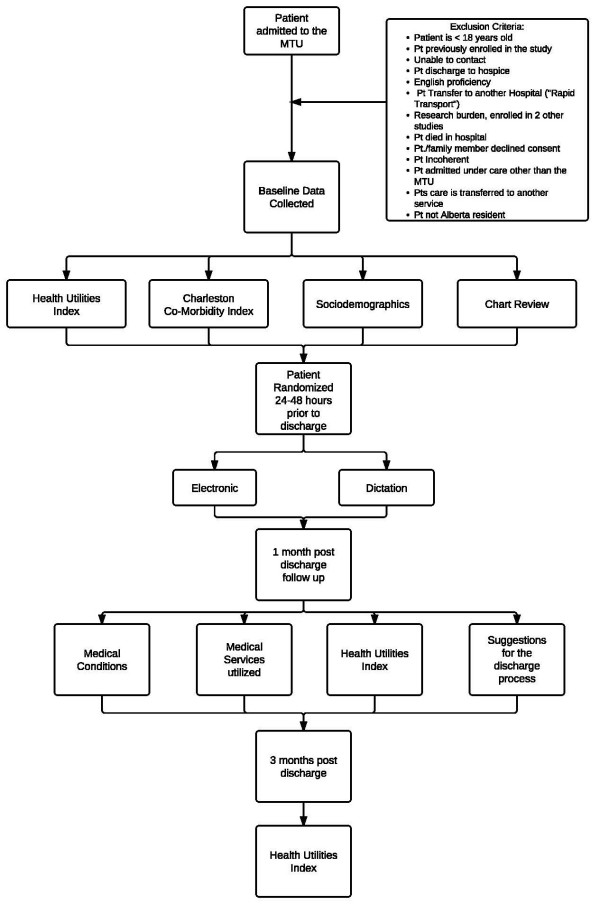
Flowchart of the protocol for the seamless transfer-of-care study.

Upon admission to the MTU, the research team will approach the patient for consent and will collect baseline data including socio-demographic information, co-morbidities (using the Charlson co-morbidity measurement method [21]), and current health status using the Health Utilities Index (HUI) [22,23]. A chart review will be conducted on each patient. This information will be collected on a secure, web-based program that was developed by the University of Calgary’s new Clinical Trial Research Unit (CRU). The CRU has also generated a web-based method for random allocation of patients to intervention *versus* control. Randomization will be requested from the investigators, and will be entirely concealed from the researchers. Randomization will be performed within 48 hours of patients leaving hospital rather than at the time of them leaving due to the nature of the MTU in which, decisions about when patients can leave often occur without much notice and residents often do not have time to complete a summary immediately given competing clinical activities. To ensure that patients will be leaving the hospital with either a copy of the completed summary produced by the TOC tool or, conversely, a hand written note for the control group, the summaries will be started by providers some time prior to the patient leaving. Figure 4 provides a summary of this process.

### Study measures

#### Baseline measures

**Patient sociodemographic characteristics** At the first study visit (baseline assessment) the patients will be presented with a brief sociodemographic questionnaire. Items included are age, sex, level of education, employment status, and admission diagnosis.

#### Chart review

Information about the patients’ admission to the MTU, comorbidities, medication changes, alcohol and substance abuse, and living arrangements will be collected from patients’ hospital charts prior to their discharge from hospital.

### Outcome measures

The primary outcome of interest for the clinical trial is the composite, at 3 months, of death or readmission to any Alberta acute-care hospital. Secondary outcomes of interest are the occurrence of post-discharge adverse events and adverse drug events at 1 month post discharge. Patient health status, meanwhile, will be assessed at baseline, 1 and 3 months using the Health Utilities Index [22,23].

The elements of our composite primary outcome (i.e., death or readmission) are identified for study because these are recognized to be major events that we are ultimately trying to prevent through safer health care. The 3 month time frame is felt to be most relevant, because it is short enough to potentially relate to discharge communications, but also long enough after discharge to permit some events to accrue. The primary outcomes of interest will be assessed through existing linkages to hospitalization data from the Alberta Health Services Health Outcomes Group (for readmission to acute care hospitals), and to the Alberta Vital Statistics for post-discharge mortality. Such an approach to post-discharge outcome ascertainment avoids the shortcoming of unreliable patient self-report on readmission, and the potential challenges associated with determining vital status of patients that we are unable to reach by telephone.

The outcomes of post-discharge adverse events and adverse drug events will be determined using the method described by Forster and colleagues [24,25]. At 1 month post discharge, a trained investigator, blinded to patient group assignments, will administer a telephone survey using a survey tool that takes approximately 15 minutes to complete to determine the post-hospital course for all consenting patients. This will allow us to determine readmission/physician visits/emergency department visits, and whether an adverse event or adverse drug event occurred. Two Royal College certified internists or College-certified family physicians, also blinded to patients’ group assignments, will independently rate the type, severity, preventability and ameliorability of all possible adverse events. Adverse events will be classified into the following types: 1. adverse drug events; 2. procedure-related injury; 3. nosocomial infection, 4. care-related fall, 5. therapeutic error, 6. diagnostic error or 7. other types. Further information on this validated multi-step method is available elsewhere [24,25].

#### Health utilities index

Patient health status and health-related quality of life (HRQOL) will be measured using the Health Utilities Index Mark 3 (HUI3) [22,23]. HUI3 assesses a full range of health among diverse groups of patients and reflects co-morbidities, and has been widely used in every major Statistics Canada population health survey in Canada since 1990 [23]. Additionally, the HUI3 can be converted to a utility score; the required input to calculate the cost per QALY gained. During the 1 and 3 months post-hospital stay, a trained investigator, blinded to patient group assignments, will administer a telephone version that takes approximately 15 minutes to complete.

#### Economic evaluation

The economic evaluation will have two primary outcomes: the cost per life and the cost per hospital readmission avoided using the TOC communication tool compared to traditional dictation. A secondary outcome will be the cost per QALY gained using the web-based TOC communication tool compared to traditional dictation. All outcomes (clinical events and utility scores) will be assessed through the clinical arm of the RCT. The cost of the TOC communication tool will be calculated from corporate financial data including the cost of development, implementation, integration and training. The cost of dictation (for type-written dictated discharge summaries the usual mode of discharge summary creation in the absence of this discharge tool) will be estimated based on physician time, transcription costs, and dissemination costs. Costs of on-going care will be estimated from administrative data including ER visits, physician visits and hospitalization. Total costs of care and outcome events will be calculated for each arm of the trial. For each objective, the cost difference between arms will be divided by the benefit difference to result in cost to benefit ratio.

### Sample size calculation and anticipated patient enrollment

As previously mentioned, a recent pilot done by the W21C team demonstrated that patients discharged from the MTU had a 23% probability of readmission to Calgary hospitals within 3 months (51 out of 219 discharged patients assessed in a pilot chart review). From this, we estimate an absolute event rate of 25% in the control group for the composite of death or readmission, and have designed our study to detect an approximately 25% relative reduction (i.e., a 6.25% absolute reduction) in the readmission rate in the intervention group. Therefore, using p_1_=0.25, and p_2_=0.1875, with alpha=0.05 and beta=0.80 in our two-tailed sample size calculation, we will require 686 patients per group. Our research plan is to enroll 700 patients per group, allowing for a safety margin for attrition. Each of the three teams usually processes an average of 2 to 4 discharges per day. As such, we anticipate that we will complete enrollment of 1400 subjects within an 18 month period and full assessment of outcomes and follow-up surveys in a 2 year period. For the secondary endpoints of post-hospital adverse events and adverse drug events, we will have adequate statistical power given that these adverse events are known to occur frequently, in approximately one quarter of patients discharged from acute-care hospitals [1,2]. The sample size is appropriate to assess patient health status measured by HUI3 to compare two independent means for a parallel trial design, 697 patients in each group are needed to detect a clinically important difference (CID) in HUI3 overall score of 0.03 (SD=0.20).

### Data analysis

The baseline characteristics (age, sex, principal diagnosis categories, and comorbididy conditions) of intervention and control subjects will be recorded and compared between groups using Chi-square tests or Fisher Exact tests where appropriate. We will also use t-tests for comparison of continuous variables. The same statistical tests will be used to compare outcomes between the two groups. Chi-square and Fisher exact tests will be used to compare the frequency of dichotomous outcomes (i.e., death or readmission, adverse events, adverse drug events). For the subset of patients who are readmitted in the two groups, we will use t-tests to compare the time-to-readmission of patients and patient health status (HUI3 overall scores) in the intervention *versus* control groups. A Kaplan-Meier analysis with log-rank test will be used to perform a time-to-event analysis in all subjects. For the economic evaluation, total costs of care and outcome events will be calculated for each arm of the trial. For each outcome, the cost difference between arms will be divided by the benefit difference to result in cost to benefit ratio.

The analysis will be performed by intention-to-treat. Patients will be analyzed in the group to which they were randomized, even if the summary of their hospital stay is generated using a communication method that differs from their group assignment. CONSORT reporting guidelines ( http://www.consort-statement.org) will be used to diligently track and report treatment crossovers and losses to follow-up through a progressive flow-chart. We will work diligently during the course of the study to limit the frequency of such occurrences. This will be achieved by a system of clear labeling of patient charts immediately after random allocation so that the care team will be aware of the assigned summary of care communication method. Research associates affiliated with the study will also be present among the clinical care team daily to reinforce the issue of compliance to patient assignments.

## Discussion

This paper outlines the study protocol for a sufficiently powered randomized controlled trial, incorporating an economic evaluation, looking at an electronic communication TOC tool developed through the partnership between Alberta Health Services, the University of Calgary, and the W21C. Since the tool is designed to transfer standardized, appropriate and accurate information in a timely manner, the study will provide evidence on how efficacious the tool will be at reducing hospital readmission, death after discharge, adverse events, adverse drug events, improving patients’ health status and its cost-effectiveness.

Randomized controlled trials (RCT) are regarded as the gold standard for clinical research assessing the efficacy of an intervention. Unlike many of the pre-post studies identified in our previously-mentioned systematic review, the present RCT is a parallel design that provides for an unbiased estimate of the effects of the intervention. Another key aspect of the present RCT is that allocation will be concealed.

Despite the obvious benefits of the parallel design with randomization, there are also some challenges. Most notably, our study design involves trade-offs between the benefits of randomizing individual patients versus the potential contamination of providers’ discharge practices when they are dealing with both the intervention and control group patients. A cluster-randomized design was considered as an approach for dealing with the latter concern. Cluster randomization has become popular in medical studies in the past two decades [26-28]. If a cluster-randomized trial design had been chosen, each individual medical team would have been allocated to either intervention or control, instead of individually allocating patients. While using a cluster-randomization design can diminish costs and lower experimental contamination (for example, the use of the electronic discharge template by a provider to guide a dictated summary in the usual care group), this method is less efficient than individual-randomized trials as members within a cluster tend to be more similar to one another than randomly allocated participants [26-28]. To compensate for the inefficiency of cluster-randomization, a larger sample size is required to meet statistical significance [28]. Furthermore, there is potential for there to be between-service differences that would affect the internal validity of the study and the comparability of the intervention and control groups. Balancing these competing concerns, and in dialogue with funding agency peer reviewers, we opted for the randomization of individual patients to intervention and control to maximize the benefits of comparability between groups.

Other challenges inherent to this study relate to acute-care physician preference and familiarity with one method for generating hospital summaries over the other, and community-care physician software incompatibilities. In a few instances, acute-care physicians may have or will develop a preference of using one system over the other. This may result in a given physician not following the patient’s assigned randomization, resulting in the exclusion of the patient and a loss of that randomization. We expect to minimize this problem by continually educating and informing the physicians of the study and the importance to adhere to the randomization assignments, as well as maintaining a strong presence throughout the MTU. More so, some community-care physicians may experience accessibility issues to the TOC summaries electronic records due to software program incompatibilities. While we foresee this as a current problem, we hope that this study will encourage the implementation of compatible programs.

This study will be one of the first to have sufficient statistical power to definitively examine the important clinical outcomes of readmission, mortality and adverse events after patients leave an acute-care facility using an electronically-based TOC communication tool. The tool that we are assessing has the potential to ameliorate a vulnerable period in health care, thus enhancing patient safety and quality of care through better communication at times of transfers of care. The tool has already been pilot tested in our jurisdiction, and through this RCT is being evaluated more broadly. This study will inform our own and other health systems globally on the potential efficacy and cost implications of such electronic communication tools. Given the widespread demand and growing investments into such electronic tools (e.g., President Obama’s $19.2 billion investment into electronic health information technology [29]) the implications of such research are truly far reaching.

## Abbreviations

W21C: Ward of the 21^st^ Century; MTU: Medical Teaching Unit; CRU: Clinical Research Trial Unit; RCT: Randomized Controlled Trial.

## Competing interests

There are no competing interests.

## Authors’ contributions

OB – Paper writing; SM – Paper writing, and Co-Principal Investigator; H-LJ – Co-investigator; FW – Co-investigator; O’BM – Co-investigator; WD – Co-investigator; FA – Co-investigator; GW – Principal Investigator. All authors read and approved the final manuscript.

## Author's information

Barbara M Okoniewska and Maria J Santana Co-First Authors on this paper.

## Pre-publication history

The pre-publication history for this paper can be accessed here:

http://www.biomedcentral.com/1472-6963/12/414/prepub
